# A Review of Some Methods of Crowning Teeth

**Published:** 1904-06

**Authors:** George F. Grant

**Affiliations:** Boston, Mass.


### A REVIEW OF SOME METHODS OF CROWNING TEETH.

BY GEORGE F. GRANT, D.M.D., BOSTON, MASS.

It is not intended by this article to lay down any special system of making crowns, but rather to consider and compare results of some systems which have come under my observation, in a practical way, at a considerable period of time after their insertion. In this connection we will consider porcelain crowns only. There was a time when they were called pivot teeth; later they were called dowel teeth; then crowns. When they took on this new title our troubles multiplied tenfold, because we tried to do so many needless things.

First, the root must be banded to insure greater strength, so that, with the band and pin together, a crown was supposed to be secured for all time. You then had a combination five times the strength of its weakest part,—to wit, the tooth. To prevent fracture of the porcelain all sorts of expedients were resorted to, until many so-called porcelain crowns could be better described as gold teeth with porcelain inlay.

The Logan has been quite extensively used, but has to my mind one great defect,—viz., the fixed position of the pin in the crown is often fatal to the proper adaptation in many cases, unless the root is so greatly weakened by enlarging the canal to accommodate the pin that, when all is completed, there remains the danger of a split root.

It would be interesting to know just the percentage of success of the Logan crowns. It is only just to say that my remarks upon them are based entirely upon observation of such as have come under my care for replacement, as I could never feel sufficient confidence in them to justify their use. This feeling of doubt has been greatly strengthened by the large percentage of cases in which the roots have been found in almost hopeless condition, either through fracture or greatly impaired strength, caused by too much reaming of the canal in the original setting of the crown.

At this time it is well to say that if you wish to lay up comfort for the future, and would avoid one of the pitfalls of the whole science, discard every material for posts except platinized gold or platino-iridium. All others will in time cause you to regret that you are still alive, for you may be sure that they will stretch or bend or corrode, or do anything that they should not do, and for that sin of small economy you will pay dearly.

The porcelain and platinum banded crown, in which the tooth and band are fused to the pin with high-fusing body, is very strong at all points; in fact, is least liable to fracture of any crown made, one point only being likely to give trouble or impair its appearance,—that is, that the enamel is very likely to flake from the band, probably caused by vibration in use. This is likely to happen after a year or two of wear. You know that the proper time to judge a crown is about the fifth year of wear.

It is by no means certain that with a good healthy root the band is not rather a doubtful factor. I believe that, when there are conditions even fairly good, it is better to do without the band and depend upon a perfect joint at or near the gum margin. This can always be assured by placing a piece of pure gold about No. 60, or ribbon gold if preferred, upon the prepared root, perforating and passing the post through it into the root. If the perforation in the plate or foil is slightly smaller than the post before it is pushed through, you may withdraw plate and post together in their proper relation, and, holding the post by the end, a little solder and a Bunsen flame unite them in a moment. Replace upon the root and mallet the gold as you would condense a filling, holding the post firmly meanwhile, and you may be certain that your cast made from an impression taken with this base and pin in position will give you all that is needed as a base upon which a perfect fitting crown can be adjusted.

This is not new, but only brought in as an illustration of one of the most successful and durable forms of crown,—one which

I left for a time to try banded crowns, and the one to which I return with confidence and a feeling of security.

About fifteen years ago I designed a form of removable crown which has proved a great success. It was suggested to my mind by the difficulty of replacing the porcelain facing, which *would* break. The plan was a square tube or socket into which the post (square also) was accurately fitted. The socket with the post inserted was first set in cement in the root, the post withdrawn after the socket was secure and levelled to the surface of the root. The post was then passed through the perforated gold base as described above, and all completed as by the first process.

A square or triangular post is always best, as there can be no danger of loosening by rotation, which I find is a greater factor in displacing of crowns than is generally considered.

A crown so constructed can be removed at any time by means of a strong, straight pull, usually by a ligature of strong silk, which will injure nothing. I have made such crowns in duplicate for patients who were likely to need a repair in places where attention could not be readily obtained, or in any case a simple direction would simplify the situation.

Though the crown was held simply by perfect contact of the sides of the post with the tube, I have never heard of one becoming loose; in fact, it requires quite a considerable force for its removal.

It is in order to say that this was before the day of the detachable facing. I find that even now it is easier to put a new facing on the old base than to find a sufficient number of detachable facings from which to make a suitable selection.

The best thing about the band crown is that it can be so efficiently applied to cases in which the post cannot be used. Many times it is imperative that crowns be supplied where the natural crown has been lost through fracture or so reduced in length by abrasion as to require it.

As it often happens that such teeth are "full of life," the process of devitalizing before crowning is undesirable, in fact, sometimes next to impossible. The way out of this difficulty seems to lie through the band crown. To illustrate, I will describe an operation which helped me out of a difficulty and made a patient happy for years. The case was one so commonly met with when the only teeth remaining in the mouth were the six anterior teeth of the lower maxilla. An artificial upper set, also lower bicuspids and molars, had been worn for some years. The difficult feature was that the six natural teeth, though healthy and firm, were so short that it was impossible to restore the face to its normal appearance unless the teeth could be lengthened. So I decided that a little experiment could do no harm. With this end in view six crowns were fitted, retained entirely by the band of each accurately fitted after slightly bevelling the anterior face of each tooth to give a good gum line. These crowns had rather a shallow seat, still the band of each was so fitted that each would stand up without cement, though of course they were finally cemented. The prosthetic work was completed to conform to the new condition established by the lengthened lower teeth. This was done in 1895. This autumn (1903) the patient desired some new plates, so that I had the opportunity of examining the six crowns. They were perfectly firm after the eight years of service, some part of which time they must have received the whole burden of mastication, as the bicuspids and molars of the lower plate had settled far below the possibility of occlusion with the upper teeth (a good argument for the use of the banded crown); for, supposing those teeth to have been devitalized in order to use posts, it is probable that they would have all loosened before this time, as the patient was at least fifty years of age at the time of the operation.

It has been my practice for many years to use a crown with band and without a post in anterior teeth, whenever devitalization has not been effected, for the reason that in all cases where it can be done upon the live stump the danger of the band coming into sight after a year or two does not exist. It is only the devitalized root that lengthens in that way, and it is especially true in the mouths of patients of mature age, say thirty-five or beyond.

Such crowns are easily repaired without injury to the stump, for if the porcelain breaks while the band holds, you have only to open the band on the palatal side to remove it. You also have the evidence of an accomplished fact that no chain can be stronger than the weakest link.

Along the lines of experience in retaining crowns by the narrow band has perhaps been built up the new system of jacket crowns, the basic principle of which is good, the conservation of the tooth-pulp.

